# Why do plasmids manipulate the expression of bacterial phenotypes?

**DOI:** 10.1098/rstb.2020.0461

**Published:** 2022-01-17

**Authors:** Kathryn Billane, Ellie Harrison, Duncan Cameron, Michael A. Brockhurst

**Affiliations:** ^1^ Department of Animal and Plant Sciences, University of Sheffield, Sheffield S10 2TN, UK; ^2^ Division of Evolution and Genomic Sciences, School of Biological Sciences, University of Manchester, Manchester M13 9PT, UK

**Keywords:** horizontal gene transfer, plasmid, parasitism, mutualism

## Abstract

Conjugative plasmids play an important role in bacterial evolution by transferring niche-adaptive traits between lineages, thus driving adaptation and genome diversification. It is increasingly clear, however, that in addition to this evolutionary role, plasmids also manipulate the expression of a broad range of bacterial phenotypes. In this review, we argue that the effects that plasmids have on the expression of bacterial phenotypes may often represent plasmid adaptations, rather than mere deleterious side effects. We begin by summarizing findings from untargeted omics analyses, which give a picture of the global effects of plasmid acquisition on host cells. Thereafter, because many plasmids are capable of both vertical and horizontal transmission, we distinguish plasmid-mediated phenotypic effects into two main classes based upon their potential fitness benefit to plasmids: (i) those that promote the competitiveness of the host cell in a given niche and thereby increase plasmid vertical transmission, and (ii) those that promote plasmid conjugation and thereby increase plasmid horizontal transmission. Far from being mere vehicles for gene exchange, we propose that plasmids often act as sophisticated genetic parasites capable of manipulating their bacterial hosts for their own benefit.

This article is part of the theme issue ‘The secret lives of microbial mobile genetic elements’.

## 1. Introduction

Plasmids are semi-autonomous, self-replicating, non-chromosomal DNA elements that are commonly present in bacterial genomes [1]. Many bacterial genomes contain multiple plasmid replicons [2,3], and plasmids have been discovered in the genomes of diverse bacterial taxa from a wide variety of ecological niches, including environmental and clinical settings [4,5]. Plasmid genes can be divided into those encoding either backbone or accessory functions [1,6]. The backbone genes encode plasmid functions, including replication and maintenance, whereas the accessory genes encode non-plasmid functions of potential utility to the bacterial host cell [1,7].

Some plasmids enable the transfer of accessory genes between bacterial strains and species, even between phylogenetically distant lineages [8]. Horizontal gene transfer (HGT) is thus a major driving force in the evolution of bacteria and has contributed significantly to the genomic and ecological diversification of bacterial taxa [9–12]. Plasmid accessory genes encode a wide range of ecological functions, including resistance to toxins, metabolic and catabolic capabilities, and production of virulence factors and anticompetitor toxins [13,14]. Plasmids thus enable their bacterial hosts to adapt to environmental stresses, such as antibiotics and toxic metals, or to colonize new niches, for example, through the exploitation of novel substrates or new hosts [8,15,16]. The huge number and diversity of accessory genes creates a vast pool of genetic variation, enabling bacteria to undergo rapid evolutionary innovation [8,17]. Given this important role in HGT, it is understandable, therefore, that most studies of the ecological and evolutionary impact of plasmids have focused on these accessory functions.

It is becoming increasingly clear, however, that besides the accessory gene functions they encode, plasmid acquisition alters the expression of a wide range of bacterial phenotypes [11,16,18]. These effects of plasmid carriage have typically been studied as the underlying causes of fitness costs because, at least in the laboratory, plasmid acquisition is frequently associated with reduced growth of plasmid-bearers compared with plasmid-free cells [11]. Costly side effects of plasmid carriage are thought to include: induction of SOS responses, cytotoxic gene products, disruption of cellular homeostasis, and the energetic burden of replicating, transcribing and translating new genetic material [11,19].

Nonetheless, plasmids have also been shown to cause differential expression of chromosomal genes, altering the expression of a wide variety of bacterial traits in ways that do not always appear straightforwardly maladaptive. Indeed, there is growing evidence to suggest that, in some cases, these plasmid-mediated alterations to the bacterial phenotype may have niche-adaptive fitness consequences that may well be missed in highly simplified laboratory environments [18]. Plasmid manipulation of bacterial gene regulation could, therefore, play an important role in the relationship between plasmids and their bacterial hosts and, moreover, could mediate the fitness effects of plasmid acquisition.

In this review, we argue that the effects that plasmids have on the expression of bacterial phenotypes may often represent plasmid adaptations, rather than mere deleterious side effects. As self-replicating biological entities, plasmids are capable of evolving adaptations to increase their own fitness. A plasmid's fitness can be defined as the sum of its vertical and horizontal replication (i.e. at bacterial cell division and plasmid conjugation events, respectively). As such, the fitness interests of plasmids need not necessarily always be aligned to those of the bacterial host cell. We begin by summarizing findings from untargeted omics analyses, which give a picture of the global effects of plasmid acquisition on host cells. Thereafter, because many plasmids are capable of both vertical and horizontal transmission, we distinguish plasmid-mediated phenotypic effects into two main classes of potential fitness benefit: (i) those that promote the competitiveness of the host cell in a given niche and thereby increase plasmid replication through vertical transmission, and (ii) those that promote plasmid conjugation and thereby increase plasmid replication through horizontal transmission.

## 2. What is the ‘omic’ footprint of plasmid acquisition upon the host cell?

Omics methods can provide an untargeted global view of the impact of plasmid acquisition on the bacterial cell. Transcriptomics, proteomics and metabolomics have each been used to compare plasmid-carrying cells with plasmid-free cells. These studies reveal extensive variation between plasmid–host pairings, in terms of both the degree of alteration caused by the plasmid and the range of cellular functions that are affected (table 1). Whereas some plasmids affect the expression or translation of several hundreds of genes and many diverse functions, other plasmids have much more limited effects upon their host cell [15,20,21].

**Table 1.** Bacterial cellular functions differently expressed following plasmid acquisition, compiled from untargeted proteomic, transcriptomics and metabolomics studies.

function	bacteria	plasmid	reference
metabolism	*Escherichia coli* DH10B, *Escherichia coli* AR060302, *Salmonella enterica* SL317, *Salmonella enterica* SL486, *Salmonella enterica* MH16125, *Shewanella oneidensis* MR-1	A/C2	[24]
amino acid metabolism	*Pseudomonas aeruginosa*	pBS228, Rms149, pAKD1, pAMBL1, pAMBL2, pNUK73	[20]
	*Pseudomonas putida *KT2440**	pCAR1	[23]
	*Pseudomonas putida *KT2440*, Pseudomonas aeruginosa *PAO1*, Pseudomonas fluorescens *Pf0-1**	pCAR1	[22]
nucleotide metabolism	*Pseudomonas putida *KT2440**	pCAR1	[23]
	*Pseudomonas aeruginosa *PAO1*, Pseudomonas fluorescens *Pf0-1**	pCAR1	[22]
energy metabolism	*Pseudomonas aeruginosa*	pBS228, Rms149, pAKD1, pAMBL1, pAMBL2, pNUK73	[20]
	*Pseudomonas putida *KT2440*, Pseudomonas aeruginosa *PAO1*, Pseudomonas fluorescens *Pf0-1**	pCAR1	[22]
	*Pseudomonas putida *KT2440*, Pseudomonas aeruginosa *PAO1*, Pseudomonas fluorescens *Pf0-1**	pCAR1	[21]
	*Escherichia coli* DH10B, *Salmonella enterica* SL317,	A/C2	[24]
carbohydrate metabolism	*Pseudomonas aeruginosa*	pBS228, Rms149, pAKD1, pAMBL1, pAMBL2, pNUK73	[20]
	*Pseudomonas putida *KT2440*, Pseudomonas aeruginosa *PAO1*,*	pCAR1	[22]
	*Pseudomonas putida *KT2440**	pCAR1	[23]
nitrogen metabolism	*Pseudomonas aeruginosa*	pBS228, Rms149, pAKD1, pAMBL1, pAMBL2, pNUK73	[20]
lipid metabolism	*Pseudomonas aeruginosa*	pBS228, Rms149, pAKD1, pAMBL1, pAMBL2, pNUK73	[20]
	*Pseudomonas putida *KT2440*, Pseudomonas aeruginosa *PAO1*, Pseudomonas fluorescens *Pf0-1**	pCAR1	[22]
respiration	*Pseudomonas putida *KT2440*, Pseudomonas aeruginosa *PAO1*, Pseudomonas fluorescens *Pf0-1**	pCAR1	[21]
	*Salmonella enterica* MH16125, *Shewanella oneidensis* MR-1	A/C2	[24]
secretion systems	*Pseudomonas aeruginosa*	pBS228, Rms149, pAKD1, pAMBL1, pAMBL2, pNUK73	[20]
Type-III	*Pseudomonas aeruginosa*	pBS228, Rms149, pAKD1, pAMBL1, pAMBL2, pNUK73	[20]
	*Salmonella enterica* SL317, *Salmonella enterica* SL486, *Salmonella enterica* MH16125	A/C2	[24]
Type-VI	*Pseudomonas aeruginosa*	pBS228, Rms149, pAKD1, pAMBL1, pAMBL2, pNUK73	[20]
	*Acinetobacter baumannii*	pAB5	[25]
	*Pseudomonas putida *KT2440**	pCAR1	[23]
signalling	*Pseudomonas putida *KT2440*, Pseudomonas aeruginosa *PAO1*, Pseudomonas fluorescens *Pf0-1**	pCAR1	[22]
translation and transcription	*Pseudomonas aeruginosa*	pBS228, Rms149, pAKD1, pAMBL1, pAMBL2, pNUK73	[20]
	*Pseudomonas putida *KT2440*, Pseudomonas aeruginosa *PAO1*, Pseudomonas fluorescens *Pf0-1**	pCAR1	[22]
motility	*Pseudomonas putida *KT2440**	pCAR1	[23]
	*Pseudomonas putida *KT2440*, Pseudomonas fluorescens *Pf0-1**	pCAR1	[22]
	*Pseudomonas putida *KT2440*, Pseudomonas aeruginosa *PAO1*, Pseudomonas fluorescens *Pf0-1**	pCAR1	[21]
	*Salmonella enterica* SL317, *Salmonella enterica* SL486, *Salmonella enterica* MH16125	A/C2	[24]
biofilm formation and adherence	*Acinetobacter baumannii*	pAB5	[25]
	*Salmonella enterica* SL317, *Salmonella enterica* SL486, *Salmonella enterica* MH16125	A/C2	[24]
TCA cycle	*Pseudomonas putida *KT2440**	pCAR1	[23]
	*Pseudomonas putida *KT2440*, Pseudomonas aeruginosa *PAO1*, Pseudomonas fluorescens *Pf0-1**	pCAR1	[21]
	*Escherichia coli* DH10B, *Shewanella oneidensis* MR-1	A/C2	[24]
iron acquisition	*Acinetobacter baumannii*	pAB5	[25]
	*Pseudomonas putida *KT2440**	pCAR1	[23]
	*Pseudomonas putida *KT2440*, Pseudomonas aeruginosa *PAO1*, Pseudomonas fluorescens *Pf0-1**	pCAR1	[22]
	*Salmonella enterica* SL486, *Salmonella enterica* MH16125, *Shewanella oneidensis* MR-1	A/C2	[24]
transporters	*Acinetobacter baumannii*	pAB5	[25]
	*Pseudomonas aeruginosa*	pBS228, Rms149, pAKD1, pAMBL1, pAMBL2, pNUK73	[20]
	*Pseudomonas putida *KT2440**	pCAR1	[23]
	*Pseudomonas putida *KT2440*, Pseudomonas aeruginosa *PAO1*, Pseudomonas fluorescens *Pf0-1**	pCAR1	[22]
	*Salmonella enterica* MH16125	A/C2	[24]

In transcriptomic studies, the percentage of differentially expressed chromosomal genes ranges from 0.59 to 20% across diverse plasmid–host interactions [15,20]. This typically includes both up- and downregulation, and where very large numbers of chromosomal genes are affected, is often linked to the plasmid altering the expression of chromosomal regulators. For example, Coulson *et al*. [15] demonstrated that two plasmid-encoded transcriptional regulators affected expression of 18% of the bacterial genome by altering expression of 31 chromosomal regulatory genes, including transcriptional regulators, sigma factors and an anti-termination regulator [15]. Similarly, Shintani *et al*. [22] showed that the acquisition of pCAR1 affected host transcriptional regulators. In a related study, pCAR1 affected the expression of 463 (8.08%) conserved open reading frames (ORFs) in *Pseudomonas putida* KT2440, several of which are involved in translation, transcription and DNA replication cellular processes [21]. Plasmid acquisition can also lead to very large fold-changes in the expression of specific chromosomal genes. For example, in *P. putida* KT2440, acquisition of the plasmid pCAR1 led to 100–200-fold upregulation of the chromosomal gene encoding the efflux system MexEF-OprN (161.8-fold change for MexE, 186.5-fold change for MexF and 113.0-fold change for OprN) [21,22] resulting in a 70-fold increase in the concentration of the MexF protein in the cell (PP_3426) [23].

Chromosomal genes differentially expressed upon plasmid acquisition are involved in a wide variety of bacterial cellular functions. These most commonly include metabolism, respiration, secretion systems, signalling, translation and transcription, motility, the tricarboxylic acid (TCA) cycle and iron acquisition (table 1). While these differentially expressed functions may be common across diverse bacterium–plasmid pairings, the specific genes affected tend to differ. Metabolic pathways altered by plasmid acquisition include amino acid and nucleotide metabolism, and metabolism of energy sources, carbohydrates, nitrogen and lipids [20–24,26]. The direction of the effect of plasmid acquisition upon the expression of secretion systems tends to vary by secretion system, such that Type-III (T3SS) and Type-IV (T4SS) secretion systems are usually upregulated, whereas Type-VI (T6SS) secretion systems are usually downregulated in plasmid carriers, though not exclusively [20,23–26]. All of these secretion systems can have a variety of functions, but generally T3SS and T4SS contribute to bacterial virulence, with an added functional role in conjugation for T4SS [27]. By contrast, T6SS secretion is involved in bacterium–bacterium communication and interaction, including toxin-mediated killing of competitors [27]. Downregulation of genes required for the flagellar complex may account for observed reduction in motility for plasmid-bearers in some cases [21,23]. Other notable bacterial functions affected by plasmid acquisition include surface polysaccharides (e.g. PNAG) and adhesion-related functions involved in biofilm formation, which, for example, in the case of *Acinetobacter baumannii* and *Salmonella enterica*, were downregulated in plasmid-bearers [24,25].

Comparative studies where the same plasmid is introduced into diverse bacterial strains or species reveal that a given plasmid can have very different transcriptional effects in different host backgrounds. For example, the A/C2 plasmid causes downregulation of pathogenicity islands in *Salmonella* hosts, but primarily affects metabolism in *Escherichia coli* strains and *Shewanella oneidensis*. Metabolic functions affected in *E. coli* included: upregulation of 2-carbon and fatty acid metabolism, glycolate metabolism and glycoxylate cycle, amino acid degradation and downregulation of amino acid biosynthesis [24]. Very few functions were affected consistently by A/C2 acquisition across all bacterial hosts. Upregulation of genes involved in oxidation/reduction reactions, cellular metabolism and metal cofactor binding occurred in all hosts, while only two genes were universally downregulated, *qacE*Δ*1* for a quaternary ammonium compound-resistance protein and *sul1* a sulfonamide-resistance dihydropteroate synthase [24]. A comparative study of the PCAR1 plasmid in three different *Pseudomonas* host species (*P. putida* KT2440, *Pseudomonas aeruginosa* PAO1 and *Pseudomonas fluorescens* Pf0-1) showed large differences in the extent of differential expression across species: 15.3% of KT2440 genes, 2.7% of PAO1 genes and 0.7% of Pf0-1 chromosomal genes [21]. Only four genes were affected by plasmid acquisition in all three host species, including one involved in iron acquisition, and two possibly involved in acetate metabolism that were in the same operon [21,22]. Interestingly, the effect of pCAR1 carriage on transcription was most similar between KT2440 and PAO1, despite KT2440 being more closely related to Pf0-1 phylogenetically, suggesting that transcriptional effects do not scale straightforwardly with genetic similarity of the host in this case.

Alternatively, changes in gene regulation have been quantified for a given bacterial host carrying different plasmids: in *P. aeruginosa* PAO1, a variety of plasmids altered regulation of a few common functional groups, most prominently metabolism (of amino acid, energy production and nitrogen) and secretion systems (Type-III and Type-VI) [20]. Furthermore, 38 chromosomal genes were consistently differentially transcribed in plasmid-bearers carrying different plasmids [20]. The rest of the transcriptional profile varied, indicating that despite these similarities, each plasmid also affected the expression of distinct sets of host functions.

Metabolic analysis has shown that plasmid acquisition can alter metabolic pathways such as glycolysis, the TCA cycle and the pentose phosphate pathway in *E. coli*, corresponding to transcriptomic data from other studies [27]. Untargeted metabolic analysis using mass spectrometry showed the abundances of a large number of compounds were affected in the same way by diverse plasmids in *P. aeruginosa* PAO1. Out of the 5000 compounds that were detected, the levels of 462 compounds were altered by plasmid acquisition across the sample set, of which the abundance of 11 compounds was significantly different in plasmid carriers for four of the six plasmids, which is much higher than would be expected by chance [20]. Of particular note were altered nucleotide abundances, particularly downregulated RNA nucleotides and upregulated (or unaltered) deoxynucleotides [20]. However, relatively few compounds could be identified (1.94%), so while metabolic analysis using mass spectrometry appears promising, more studies that cover a greater diversity of bacterial species and plasmids will be needed to identify robust patterns.

The existing omics studies discussed here have some limitations. First, it is rarely confirmed that the plasmid-carrying transconjugants have not acquired other chromosomal mutations that may alter chromosomal transcription independently of the plasmid. This could be determined by curing the plasmid and confirming that transcription returns to wild-type levels, or by genome sequencing the transconjugant to confirm no additional mutations are present [28]. More studies with these additional controls would be valuable. The studies discussed here also almost exclusively focus on gammaproteobacterial hosts, and it would be useful for future studies to investigate the impact of plasmids in a broader taxonomic range of bacterial hosts outside of this well-studied clade.

The diversity of plasmids is such that it may be difficult or impossible to predict *a priori* how plasmid-encoded genes interact with bacterial regulatory networks [11,29]. We might expect that adaptive plasmid manipulation would cause relatively consistent transcriptional effects across multiple host genotypes encountered in the plasmid's recent evolutionary history. By contrast, among the few existing comparative studies, it would appear that each bacterium and plasmid pairing has a different, unique differential expression profile. However, such studies typically use a few bacterial strains isolated from different locations and habitats; meanwhile, the natural host of the plasmid is often unknown. Future studies are required, therefore, that compare the transcriptional effects of plasmids upon hosts that they coexisted with in nature within ecologically coherent communities, and thus are likely to represent the recent selective environment for the plasmid. In the studies highlighted above, although the specific genes affected may vary, groups of cellular functions commonly affected by plasmid carriage do begin to emerge, for example, bacterial metabolism appears to be the most frequently affected of these functions. While this could represent adaptive manipulation by the plasmid, an alternative hypothesis is that this could instead be a generic response of bacteria to the acquisition of plasmids, and future studies should attempt to distinguish between these competing hypotheses. In future, it will also be valuable to study how the expression of bacterial functions is affected by plasmid acquisition within the context of relevant environmental niches to better understand how plasmids shape the host bacterial phenotype and fitness in nature.

## 3. Linking altered expression of bacterial functions to plasmid fitness

Understanding the evolutionary impact of plasmid manipulation of the expression of bacterial phenotypes requires an understanding of how these different bacterial phenotypes are linked to plasmid fitness. Plasmid fitness has two main components, first, replication by vertical transmission to daughter cells, and second, replication by horizontal transmission through cell-to-cell conjugation. In the following sections, we suggest ways in which plasmid manipulation of the expression of chromosomally encoded bacterial traits could potentially affect these plasmid fitness components.

### (a) Bacterial phenotypes likely to affect plasmid vertical transmission

**Figure 1.**
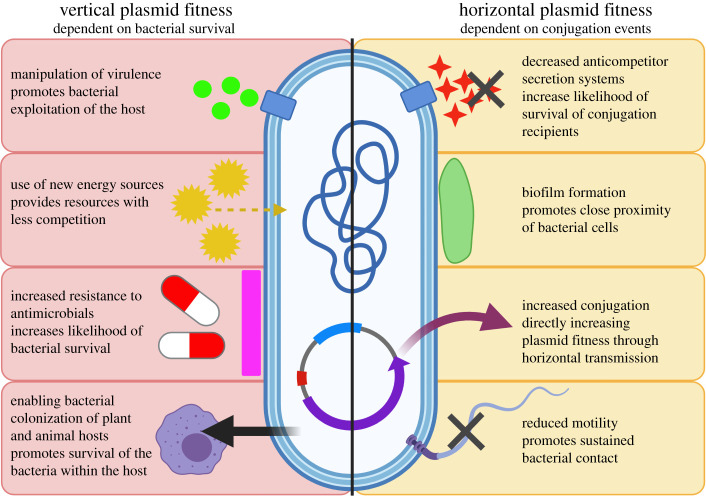
A schematic of how the bacterial phenotypes altered by plasmid acquisition could affect plasmid fitness (created in BioRender.com). We distinguish phenotypic effects according to their likely effects on the modes of plasmid inheritance, vertical from mother cell to daughter cell by replication, or horizontal from cell to cell by conjugation.

Increased vertical plasmid transmission can result from enhanced survival and/or growth of the host bacterium in a given niche. We make the distinction between plasmid fitness benefits deriving from the accessory genes encoded by the plasmid and those caused by differential expression of chromosomally encoded bacterial genes, and focus here only on the latter. To illustrate this idea, we highlight bacterial phenotypes where plasmid-induced changes in expression of chromosomal genes could cause niche-adaptive alterations benefiting both the bacterium and the plasmid. We suggest that this evolutionary strategy could be evident in plasmid manipulation of bacterial traits, including virulence, resistance to antimicrobials and metabolism, that allow bacterial cells to survive stressors or colonize new niches (figure 1).

#### (i) Increased resistance to antimicrobials

Although many plasmids encode antibiotic resistance genes, in a number of cases, plasmid acquisition has been shown to alter the expression of chromosomally encoded resistance determinants. For example, acquisition of the pCAR1 plasmid causes massive upregulation of the MexEF–OprN efflux system in a number of *Pseudomonas* host species [20]. The MexEF–OprN efflux system provides resistance to a range of antibiotics, including some quinolones, sulfonamides and chloramphenicols [21–23]. Carriage of pCAR1 is, therefore, likely to increase bacterial resistance to antibiotics without itself encoding antibiotic resistance genes, thus potentially enhancing the survival of plasmid-carrying bacterial cells (and thus the plasmid itself) in antibiotic-containing environments.

#### (ii) Alternative energy sources

The most common differentially regulated bacterial function affected by plasmid acquisition is metabolism. Often, multiple aspects of metabolism are altered (e.g. carbohydrate, energy, amino acid), with the direction of regulation often varying among bacterium–plasmid pairings, sometimes for the same functional group of genes [20]. An interesting example where a consistent effect is observed across diverse host strains is the plasmid pLL35, which causes the upregulation of bacterial anaerobic metabolism genes in phylogenetically diverse *E. coli* backgrounds [28]. Although the effect on bacterial growth is unknown, it is possible that by shifting the host cell from aerobic towards anaerobic metabolism, the plasmid may potentiate gut colonization, and thereby promote the fitness of both the bacterium and the plasmid in this niche.

#### (iii) Host colonization

Several plasmids have been shown to manipulate the expression of traits likely to enhance bacterial survival within eukaryotic hosts [30]. For example, certain extended spectrum beta-lactamase (ESBL) plasmids upregulate genes (*ompA, nha, dnaJ, arcA*) and outer membrane proteins that enhance survival of extra intestinal pathogenic *E. coli* in host serum [31,32]. The plasmid pMAR2 upregulates expression of a chromosomal adhesin in enteropathogenic *E. coli*, thus enhancing host colonization by promoting the formation of attaching and effacing lesions in intestinal epithelial cells [33]. Finally, a *Rhodoccocus equi* plasmid alters the expression of chromosomal virulence regulators promoting macrophage colonization [15] by arresting phagosomal maturation [34]. In each of these cases, by enhancing bacterial survival within the eukaryotic host, the plasmids may increase their own fitness as well as that of their bacterium in this niche.

#### (iv) Virulence

Plasmids can promote bacterial exploitation of eukaryotic hosts by altering the production of chromosomally encoded virulence factors. Several plasmids upregulate the bacterial T3SS [20,24], which delivers toxins to degrade eukaryotic cells, thus freeing up host resources for bacterial consumption. In *Chlamydia* species, a plasmid-encoded transcriptional regulator, Pgp4, controls expression of chromosomal genes required for the bacterium to exit the host infected cell in order to infect other cells, a fundamental stage in the *Chlamydia* infection cycle [35–38].

### (b) Bacterial phenotypes likely to affect plasmid horizontal transmission

Many plasmids can transfer horizontally to new host cells by conjugation. Even non-conjugative plasmids sometimes undergo horizontal transfer by piggy-backing on the conjugation machinery of other coexisting plasmids, and this can be vital to ensure their survival in the population [39]. The rate of plasmid conjugation is usually plasmid-regulated in a manner that is responsive to conditions in the host cell, such as growth stage [40]. In addition, the rate of plasmid conjugation varies across environments and, for example, can be higher on surfaces that enable higher levels of cell–cell contact than in planktonic culture [41,42]. In what follows, we highlight examples where plasmids induce changes in bacterial phenotypes that could enhance plasmid conjugation, promoting spread of the plasmid in the bacterial population or community. Because conjugation is energetically expensive to host cells and exposes them to killing by phages that bind the conjugation pilus, these phenotypic changes may be to the detriment of host cell fitness. Bacterial phenotypes that may potentially enhance plasmid horizontal transmission include manipulation of motility, biofilm formation, the T6SS and the DNA replication process (figure 1).

#### (i) Motility

Plasmid acquisition is often associated with reduced bacterial motility, sometimes caused by plasmid-mediated downregulation of the flagellar complex [21,23,24,43,44]. Cell-to-cell contact is vital for successful conjugation [41], and thus reduced motility may increase the likelihood that bacterial cells remain in contact long enough for the plasmid to undergo conjugation [45], thus potentially enhancing the horizontal transmission of the plasmid.

#### (ii) Biofilm formation

Increased biofilm formation has been reported in a range of bacterial taxa upon acquisition of conjugative plasmids [46–48]. In *Bacillus subtilis*, increased biofilm formation is mediated by a plasmid-encoded Rap protein (RapP), an intracellular response regulator involved in biofilm formation and sporulation, among other functions [48,49]. Similarly, in some strains of enteropathogenic *E. coli*, ESBL plasmid acquisition is associated with increased production of extracellular biofilm components [32]. Opportunities for plasmid conjugation are expected to be increased in spatially structured populations such as biofilms, presumably owing to increased cell-to-cell contacts, and, therefore, increasing biofilm production may well indirectly increase plasmid horizontal transmission.

#### (iii) Maintenance and transfer

Plasmid pCAR1 encodes three nucleoid-associated proteins (NAPs). NAPs are global regulators in transcriptional networks, affecting quorum-sensing systems, and bacterial metabolism [50–53]. Intriguingly, plasmids that encode NAPs are more likely to be conjugative [54], suggesting that plasmids may use NAPs to manipulate host cell regulatory networks in ways that promote horizontal plasmid fitness.

#### (iv) Altering bacterial competition

In *A. baumannii*, the plasmid pAB5 encodes a repressor that deactivates the bacterium's T6SS [55], which would otherwise kill non-kin cells by injecting them with toxins. By deactivating the host cell's T6SS, however, the plasmid ensures the survival of transconjugants, thus increasing the success of conjugation events [56] and thereby the plasmid's rate of horizontal transmission. Intriguingly, by leaving the original host cell unable to deploy its T6SS apparatus in competition with other bacteria, the plasmid may decrease its host's own fitness. This illustrates how plasmid fitness interests can conflict with the bacterial host's fitness interests. Such traits can be favoured provided that the resulting increase in horizontal plasmid replication outweighs the loss of vertical plasmid replication.

## 4. Future research directions

This review has highlighted some of the growing evidence that the relationship between plasmids and bacteria may be more subtle and manipulative than previously acknowledged. Plasmid manipulation of the expression of bacterial chromosomal genes demonstrates the breadth of parasitic and mutualistic evolutionary strategies plasmids use to maximize fitness. Future studies should consider the following directions:

— How does plasmid manipulation vary across environmental contexts? Laboratory conditions are unlikely to reveal the full extent of niche-specific phenotypic effects caused by plasmid manipulation. Some of the largest effects on bacterial functions have been seen in studies that assess fitness in macrophages or serum [15,31,32]. In macrophages the plasmid affected expression of 20% of bacterial chromosomal genes, including those that slowed phagosome maturation, a key virulence strategy for survival within the eukaryotic host. Future studies should be conducted under conditions more similar to those encountered by the bacteria in nature.
— How does plasmid manipulation vary across a broader taxonomic range of bacterial hosts? Most of the studies discussed in this review have focused on gammaproteobacterial hosts. In order to gain a fuller and more representative view of the impact of plasmids on the expression of bacterial phenotypes beyond this clade, future studies should test a far broader diversity of bacterial hosts and plasmids.
— How might integrated omics studies aid our understanding of how differential regulation leads to altered bacterial phenotypes? Untargeted omics approaches are an efficient way of obtaining the molecular underpinning of bacterial phenotype, and allow us to see nuanced effects of plasmid acquisition. There are many more metabolites than genes to encode their synthesis, and metabolic pathways are complex and adaptable [57]. It is nearly impossible to predict effects on the metabolome from the wide array of genes that may be differentially expressed upon plasmid acquisition. Therefore, an integrated, multifaceted omics approach may reveal more of the story.
— How does plasmid manipulation of bacteria evolve? One obvious route for plasmid co-option of bacterial gene regulation would be through duplication of bacterial regulatory genes onto the plasmid, followed by divergence. Plasmids (and other mobile elements) frequently acquire bacterial genes through rearrangements [58]. However, it is unclear if such an evolutionary path would be likely. Genes heavily embedded into gene networks tend to be underrepresented on mobile elements [59]. This may be explained by highly connected genes causing far higher disruption to the cell regulatory network [59]. Duplication of bacterial regulatory genes may, therefore, face more significant fitness barriers to establishment than, for example, the acquisition of an accessory trait. Alternatively, plasmid manipulation may arise through non-specific disruption of regulatory networks. Plasmid acquisition can lead to widespread, subtle (and not so subtle) shifts in bacterial gene expression [20–24]. Where these shifts benefit the plasmid, they may be acted on by selection to further embed this function. Further work will be needed to determine what evolutionary trajectories lead to the origination of plasmid regulatory manipulation.
— What are the dynamics of plasmid manipulation traits in bacterial populations and communities? The inheritance of plasmid manipulation traits is likely to differ significantly from inheritance of accessory traits. Plasmid accessory traits are typically, perhaps necessarily [59], self-contained regulatory units whereas manipulation of bacterial gene regulation is likely to be dependent and contingent upon the regulatory network(s) present in the bacterial host. Following from this, we might predict that bacterial manipulation traits may only function in a narrow taxonomic range of hosts, explaining the high variability in the breadth and extent of regulatory effects across hosts, whereas by contrast accessory genes are expected to function similarly across taxonomically diverse hosts.

## 5. Conclusion

Plasmids can have effects on bacterial phenotypes that extend beyond those conferred by the accessory gene cargo that they encode, by manipulating the expression of genes encoded on the bacterial chromosome. We identify two possible ways that such manipulation could affect plasmid fitness: first, by increasing the growth of the bacterial cell in a particular niche and thus increasing the vertical transmission of the plasmid, or second, by altering the phenotype of bacterial cells in ways that increase the likelihood of conjugation of the plasmid, thus increasing its horizontal transmission. This dichotomy highlights the potential for plasmid manipulation of bacterial phenotypes to result in both mutualistic and parasitic interaction with the bacterial host. Identifying the mechanisms of plasmid manipulation is challenging (cf. [55]) but will be essential to better understand how and why plasmid manipulation has evolved and the role it plays in the evolutionary success of plasmids.

## Data accessibility

This article has no additional data.
